# CircRNA_0000392 promotes colorectal cancer progression through the miR-193a-5p/PIK3R3/AKT axis

**DOI:** 10.1186/s13046-020-01799-1

**Published:** 2020-12-14

**Authors:** Hanchen Xu, Yujing Liu, Peiqiu Cheng, Chunyan Wang, Yang Liu, Wenjun Zhou, Yangxian Xu, Guang Ji

**Affiliations:** 1 Institute of Digestive Diseases, Longhua Hospital, Shanghai University of Traditional Chinese Medicine, Shanghai, 200032 China; 2 Department of General Surgery, Longhua Hospital, Shanghai University of Traditional Chinese Medicine, Shanghai, 200032 China

**Keywords:** Colorectal cancer, Circular RNAs (circRNAs), RNA sequencing, CircRNA_0000392, miR-193a-5p, PIK3R3

## Abstract

**Background:**

Circular RNAs (circRNAs), important members of the noncoding RNA family, have been recently revealed to play a role in the pathogenic progression of diseases, particularly in the malignant progression of cancer. With the application of high-throughput sequencing technology, a large number of circRNAs have been identified in tumor tissues, and some circRNAs have been demonstrated to act as oncogenes. In this study, we analyzed the circRNA expression profile in colorectal cancer (CRC) tissues and normal adjacent tissues by high-throughput sequencing. We focused on circRNA_0000392, a circRNA with significantly increased expression in CRCtissues, and further investigated its function in the progression of colorectal cancer.

**Methods:**

The expression profile of circRNAs in 6 pairs of CRC tissues and normal adjacent tissues was analyzed by RNA sequencing. We verified the identified differentially expressed circRNAs in additional samples by qRT-PCR and selected circRNA_0000392 to evaluate its associations with clinicopathological features. Then, we knocked down circRNA_0000392 in CRC cells and investigated the in vitro and in vivo effects using functional experiments. Dual luciferase and RNA pull-down assays were performed to further explore the downstream potential molecular mechanisms.

**Results:**

CircRNA_0000392 was significantly upregulated in CRC compared with normal adjacent tissues and cell lines. The expression level of circRNA_0000392 was positively correlated with the malignant progression of CRC. Functional studies revealed that reducing the expression of circRNA_0000392 could inhibit the proliferation and invasion of CRC both in vitro and in vivo. Mechanistically, circRNA_0000392 could act as a sponge of miR-193a-5p and regulate the expression of PIK3R3, affecting the activation of the AKT-mTOR pathway in CRC cells.

**Conclusions:**

CircRNA_0000392 functions as an oncogene through the miR-193a-5p/PIK3R3/Akt axis in CRC cells, suggesting that circRNA_0000392 is a potential therapeutic target for the treatment of colorectal cancer and a predictive marker for CRC patients.

## Background

Colorectal cancer (CRC) threatens human health as the third most common cancer worldwide [1]. In recent years, the incidence of colorectal cancer has increased annually [2]. With the continuous improvement of diagnosis and treatment, the five-year survival rate of colorectal cancer has increased, but the five-year survival prognosis is highly correlated with the stage of the disease. Patients with advanced colorectal cancer are typically accompanied by tumor metastasis, and the five-year survival rate is very low [3]. Therefore, it is urgent to further study the pathogenesis of colorectal cancer and the unknown molecular mechanism involved in tumor metastasis.

There is a large amount of noncoding RNA in the human genome, and the relationship between the existence of noncoding RNA and human diseases has always been a research hotspot, especially in malignant tumors. Circular RNAs (circRNAs) are important members of the noncoding RNA family along with microRNAs and lncRNAs. CircRNAs are characterized by their covalently closed loop structures and the absence of 3′ and 5′ ends. Based on this closed structure, circRNAs are highly stable and not easily degraded [4]. Researchers have discovered the presence of circRNAs in multiple organisms, such as yeast, mitochondria and eukaryotes, and detected more than 20,000 circRNAs in eukaryotes [5, 6]. One study reported that exon rearranged circulating transcripts were first discovered in leukemia cells, HeLa cell lines and normal human primary blood cells, and approximately 80 circRNAs were identified [7]. Since then, an increasing number of circRNAs have been identified in different tissues using high-throughput sequencing technology. The role of circRNA in the development of diseases, such as encephalopathy and tumors, has also been gradually revealed [8, 9]. The mechanism of circRNA as a competitive endogenous RNA has become a focus of cancer research. CircRNA adsorbs miRNA through the sponge action of miRNA to regulate the expression of its target genes [10, 11]. With the continuous expansion of studies on circRNAs, circRNAs have been shown to be involved in the development of almost all types of cancers [12–17]. All the studies on the relationship between circRNAs and cancer suggest that circRNAs may be novel potential biomarkers and therapeutic targets. However, since most circRNAs are still not fully characterized and the roles of circRNAs in CRC progression are still largely unknown, further research is needed to identify the circRNAs associated with CRC tumorigenesis and to elucidate their functions.

In this study, we first explored the expression profiles of circRNAs in 6 paired CRC tissues and adjacent normal tissues by using high-throughput RNA sequencing. A total of 66,855 circRNAs were detected, among which 1687 circRNAs with significant differential expression were identified after CRC tissues were compared with adjacent normal tissues. After verifying some candidate circRNAs by qRT-PCR, we found that circRNA_0000392, which originates from exons 2 to 4 of the YAF2 gene, was significantly upregulated in CRC tissues. The high expression of circRNA_0000392 was associated with pathological stage and metastasis in CRC. We then focused on circRNA_0000392 and demonstrated that inhibition of its expression could significantly attenuate the proliferation and invasion of CRC cells. More importantly, we explored the mechanism of circRNA_0000392 in the progression of colorectal cancer and found that it could act as a sponge of miR-193a-5p, thereby releasing the inhibition of PIK3R3 by miR-193a-5p and promoting the phosphorylation of the AKT/mTOR signaling pathway. Our findings illustrate a new mechanism of CRC progression and provide new insights for the treatment and diagnosis of CRC.

## Materials and methods

### Patient population and clinical data

Forty pairs of CRC tissues and adjacent normal tissues were collected from patients who were diagnosed with CRC at the Longhua Hospital affiliated with Shanghai University of Traditional Chinese Medicine (Shanghai, China). Tumor and normal adjacent tissue samples were obtained during surgical treatment at the Department of General Surgery. The samples were isolated, immediately snap frozen in liquid nitrogen and stored at − 80 °C before use. All patients signed informed consent forms prior to surgery and did not receive preoperative chemotherapy or radiotherapy. This study was approved by the Ethics Committee of Longhua Hospital.

### RNA sequencing, identification and quantification of human circRNAs

Total RNA was isolated from the tissue samples using TRIzol reagent (Life Technologies, Carlsbad, CA) according to the manufacturer’s instructions. Then, we assessed the RNA integrity and DNA contamination by using electrophoresis on a denaturing agarose gel. After confirming that the RNA was intact and pure, we used the Ribo-Zero rRNA Removal Kit (Illumina, San Diego, CA, USA) and the CircRNA Enrichment Kit (Cloud-seq, USA) to remove the rRNA and enrich the circRNAs, respectively. The RNA-seq libraries were constructed using pretreated RNAs with the TruSeq Stranded Total RNA Library Prep Kit (Illumina, San Diego, CA, USA) according to the manufacturer’s instructions. The libraries were denatured as single-stranded DNA molecules, captured on Illumina flow cells, amplified in situ as clusters and finally sequenced for 150 cycles on an Illumina HiSeq™ 4000 Sequencer (Illumina, San Diego, CA, USA) according to the manufacturer’s instructions. Paired-end reads were harvested from an Illumina HiSeq™ 4000 sequencer and were quality controlled by Q30. The reads were aligned to the reference genome/transcriptome with STAR software, and circRNAs were detected and annotated with DCC software. The circBase database and circ2Trait disease database were used to annotate the identified circRNAs. The differentially expressed circRNAs between the two groups were identified using T test statistical methods.

### Analyses of circRNA-miRNA-mRNA interactions in CRC

CircRNA-miRNA interactions were predicted by popular target prediction software, including Circular RNA Interactome and RegRNA. Specific predictions for the target genes of miRNAs were based on the miRanda, miRDB, miRWalk, RNA22 and TargetScan databases. All circRNA-miRNA-mRNA networks were constructed using Cytoscape software.

### Cell culture

Human CRC cell lines (HT29, HCT116, SW480, SW837, SW48, SW620 and RKO) were purchased from American Type Culture Collection (ATCC) (Manassas, VA, USA). A normal human colon mucosal epithelial cell line (NCM460) and the 293 T cell line was obtained and preserved in our lab. HT29 and HCT116 cells were cultured in McCoy’s 5A (Gibco, Carlsbad, CA, USA), while SW480, SW620, SW48, SW837 and 293 T cells were cultured in DMEM (Gibco, Carlsbad, CA, USA). NCM460 cells were cultured in M3:10 media (INCELL, San Antonio, TX), and RKO cells were cultured with MEM (Gibco, Carlsbad, CA, USA). All culture media contained 10% fetal bovine serum and 1% penicillin. All these cell lines were maintained in a humidified atmosphere of 5% CO_2_ at 37 °C.

### Antibodies and reagents

Anti-PIK3R3 antibody (ab97862, 1:1000 dilution for immunoblotting and 1:200 for IHC) was purchased from Abcam. Anti-AKT1 antibody (#2938), anti-phospho-Akt (Ser473) antibody (#4058), anti-mTOR antibody (#2972), and anti-phospho-mTOR (Ser2448) antibody (#2971) were obtained from Cell Signaling Technology, and all antibodies were diluted 1:1000 for immunoblotting. Anti-actin (sc-1616, 1:5000 dilution), HRP-conjugated anti-mouse IgG (sc-2055, 1:5000 dilution) and HRP-conjugated anti-rabbit IgG (sc-2054, 1:5000 dilution) were purchased from Santa Cruz. Actinomycin D and crystal violet were purchased from Sigma-Aldrich (St Louis, MO, USA). RNase R was purchased from Epicentre Technologies (Madison, WI, USA).

### RNA extraction and qRT-PCR

Total RNA was extracted by using TRIzol reagent (Life Technologies, Carlsbad, CA) and then reverse-transcribed into cDNA using the SuperScript First-Strand Synthesis System (Invitrogen, Carlsbad, CA, USA). cDNA was used for qPCR performed with the SYBR Green PCR Master Mix (Applied Biosystems, Foster City, CA, USA) and gene-specific primers, and the results were normalized using β-actin or U6 as a control. PCR primers are listed in Additional file 1: Table S1.

### CircRNA RNase R resistance analysis and actinomycin D assay

SW620 and RKO cells were treated with 3 U/mg RNase R (Epicentre, WI, USA) or 2 mg/L actinomycin D (Sigma, USA) and then cultured at 37 °C. The cells were harvested at the indicated time points, and the stability of circRNA_0000392 and YAF2 mRNA was detected by quantitative real-time PCR (qRT-PCR) assay.

### Fluorescence in situ hybridization (FISH)

SW620 and RKO cells were seeded in dishes and cultured until 70–80% confluence. Then, the cells were fixed at room temperature with 4% paraformaldehyde and treated with protease K. Then, the cells were overlaid with FITC-labeled circRNA_0000392 probe (Gefanbio, China) at 65 °C for 48 h. The signals of the probe were detected by a Fluorescent In Situ Hybridization Kit (Gefanbio, China) according to the manufacturer’s protocol. Nuclei were counterstained with DAPI.

### Luciferase reporter assay

The sequences of circRNA_0000392 and the PIK3R3 3′ UTR and their corresponding mutant versions without miR-193a-5p binding sites were synthesized and subcloned into the luciferase reporter vector pmirGLO (Promega, Madison, WI, USA), and the resulting constructs were named circRNA_0000392 -WT, circRNA_0000392-Mut, PIK3R3 3′ UTR-WT and PIK3R3 3′ UTR-Mut, respectively. The plasmids were validated by sequencing and then cotransfected with the miRNA mimics or inhibitor or the corresponding negative controls. The relative luciferase activity was measured using a Dual Luciferase Assay Kit (Promega, Madison, WI, USA).

### Transwell migration and Matrigel invasion assays

A Transwell chamber (Corning, Kennebunk, ME, USA) was used for the migration assays, and a transwell chamber precoated with Matrigel was used for the invasion assays. According to the protocol, single-cell suspensions were added to the upper chambers and incubated for 24 h. Then, the cells were washed, fixed, and stained with crystal violet. Based on the crystal violet staining data, we calculated the migration and invasion rates by counting the cells in at least five random fields.

### RNA immunoprecipitation (RIP)

RIP assays were performed in SW620 and RKO cells. A total of 1 × 10^7^ cells were completely lysed by RNA lysis buffer and then incubated with RIP immunoprecipitation buffer containing magnetic beads conjugated with human anti-Argonaute2 (AGO2) antibody (Millipore, USA) or negative control mouse IgG (Millipore, USA). Proteinase K was added to the RIP sample and incubated at 55 °C for 30 min. Then, immunoprecipitated RNA was isolated and analyzed by qRT-PCR to quantify the enrichment of circRNA_0000392.

### RNA pull-down

Biotin-labeled circRNA_0000392 probe or oligo probe (GenePharma, China) were synthesized. SW620 and RKO cells were lysed with lysis buffer and incubated with specific circRNA_0000392 probes. Then, SW620 and RKO cells were lysed with lysis buffer and incubated with probe-coated beads at 4 °C overnight. The beads were washed, the RNA complexes were extracted with TRIzol (Life Technologies, Carlsbad, CA) and detected by qRT-PCR.

### Immunohistochemistry

Detection of the expression level of PIK3R3 by immunohistochemistry was performed on 5-μm thick paraffin sections of patient tissue samples. Briefly, the sections were deparaffinized and rehydrated followed by antigen retrieval using 0.01 M sodium citrate buffer (pH 6.0) at a boiling temperature for 10 min. Then, the sections were incubated with 3% hydrogen peroxide for 10 min, 5% bovine serum albumin for 1 h and primary antibodies at 4 °C overnight. The sections were incubated with secondary antibodies after washing three times with PBS. Finally, the DAB system was used to visualize the signal, and hematoxylin was used to stain the nucleus. The immunostaining images were captured using an Olympus FSX100 microscope (Olympus, Japan).

### Xenograft tumor model

BALB/c nude mice (male, 3- to 4-week-old) were injected subcutaneously with 5 × 10^6^ SW620 cells. Tumor volumes were measured with a caliper every 3 days and calculated from the length (a) and the width (b) by using the following formula: volume (mm^3^) = ab^2^/2. Thirty days after injection, the animals were sacrificed, and the excised tumor tissues were removed to further assess tumor weight and pathological staining.

### Statistical analysis

Statistical analyses were performed using GraphPad Prism 7.0 (GraphPad Software Inc., CA, USA). Student’s t-test and one-way ANOVA were used to compare differences between groups as appropriate. The correlation between groups was analyzed by Pearson correlation. ROC curve analysis was performed to evaluate the diagnostic value. Data are presented as the mean ± standard deviation (SD), and *p* < 0.05 was considered statistically significant.

### Additional methods

The cell transfection, western blot, cell proliferation, and apoptosis assays are described as the Supplementary Methods in Additional file 2.

## Results

### Identification of circular RNAs by RNA-seq analyses in human CRC

**Fig. 1:**
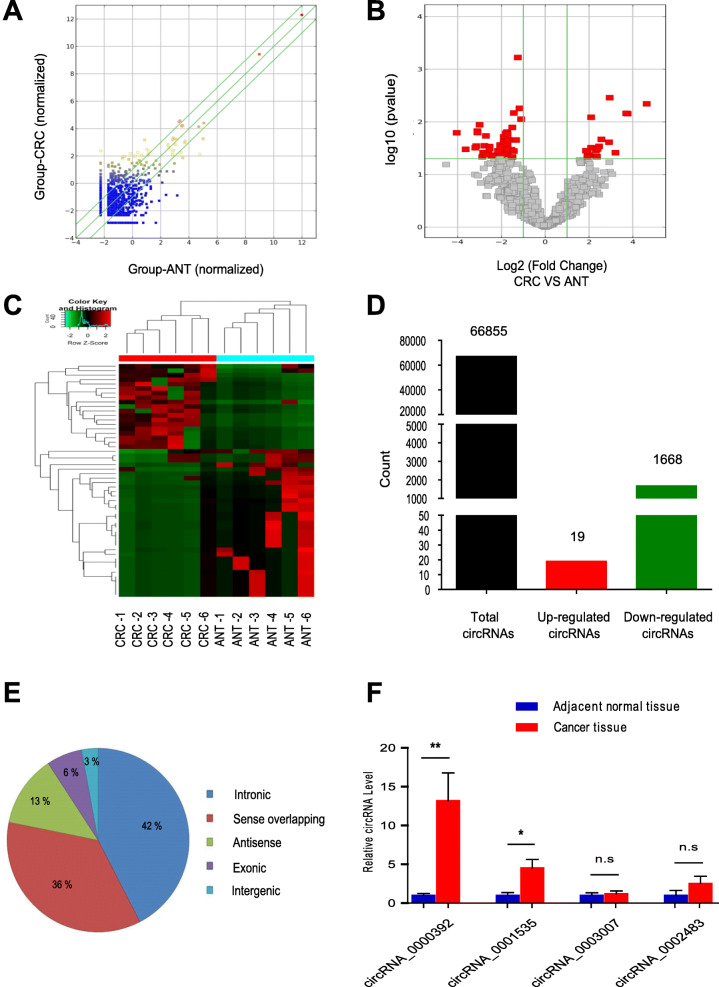
Identification of circular RNAs by RNA-seq analyses in human CRC samples. **a** The scatter plot shows the changes in circRNA expression in six paired CRC and adjacent normal tissues (ANT). CircRNAs above the top green line and below the bottom green line demonstrated a greater than 1.5-fold change between the two compared groups. **b** The volcano plot shows the expression profiling of circRNA between CRC and ANT. The vertical green lines refer to a 2.0-fold (log2 scaled) upregulation and downregulation, respectively. The horizontal green line corresponds to a *P*-value of 0.05 (−log10 scaled). The red points in the plot represent differentially expressed circRNAs with statistical significance. **c** Clustered heat map indicating differences in circRNA expression profiling between CRC and ANT tissues. **d** The number of total circRNAs identified by RNA-seq and the number of differentially expressed circRNAs. **e** CircRNAs were classified by categories. **f** Validation of the top 4 differentially expressed circRNAs in 16 paired CRC and ANT tissues by RT-qPCR. CRC, colorectal cancer; ANT, adjacent normal tissue. Data represent the mean ± SD. * *P* < 0.05, ** *P* < 0.01

To obtain the expression profiles of circRNAs and identify differentially expressed circRNAs in CRC patients, secondary sequencing was used to profile circRNA expression in paired CRC tissues and adjacent normal tissues (ANT) from 6patients with CRC. First, the scatter plot showed the differences in circRNA expression between the tumor tissues and adjacent normal tissues (Fig. 1a). Then, under the cutoff criteria of fold change > 2.0 and *P* < 0.05, the significant differentially expressed circRNAs between the two groups are presented in a volcano plot (Fig. 1b) and hierarchical cluster (Fig. 1c). In total, 66,855 circRNAs were detected in the tissue samples by sequencing, including 19 significantly upregulated and 1668 significantly downregulated circRNAs (Fig. 1d and Additional files 3 and 4). GO and KEGG analyses of the host genes of differentially expressed circRNAs are shown in Additional file 1: Fig. S1 and S2. Based on their genomic origin, circRNAs were classified as intronic, exonic, intergenic, antisense and sense overlapping circRNAs and are presented as a pie chart showing the percentage of significantly differentially expressed circRNAs in each category (Fig. 1e). We then identified the circRNAs that can function as miRNA sponges from the significantly differentially expressed circRNAs and constructed a network map using Cytoscape software (Additional file 1: Fig. S3). We overlapped the top 10 circRNAs that were significantly upregulated or downregulated (Additional file 1: Table S2) with circRNAs that function as miRNA sponges. From the overlap, we first selected 4 circRNAs and verified the circRNA-seq results in 40 paired CRC tissues and adjacent normal tissues (ANT) by qRT-PCR. As shown in Fig. 1f, circRNA_0000392 expression was significantly upregulated in CRC tissues, which was consistent with the sequencing data. Of the two, circRNA_0000392 had an approximately 25-fold change in sequencing and an approximately 13-fold change in 16 patients, indicating a more significant difference. Therefore, in this study, we will focus on the role of circRNA_0000392 in the tumorigenesis and progression of colorectal cancer.

### circRNA_0000392 is upregulated in CRC and associated with progression

**Fig. 2:**
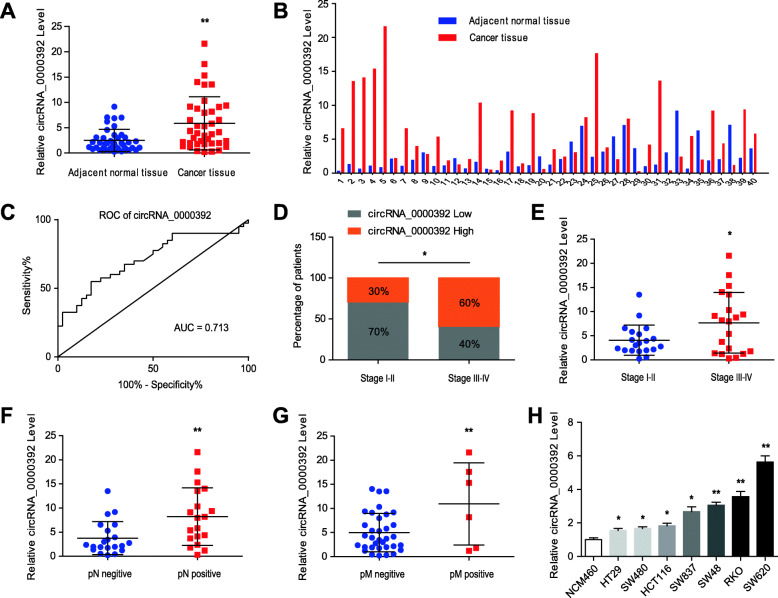
CircRNA_0000392 is upregulated in CRC and associated with progression. **a** - **b** Relative expression of circRNA_0000392 in 40 pairs of CRC and ANT tissues measured by qRT-PCR. **c** ROC curve analysis of circRNA_0000392 in CRC patients. AUC values are included in the graphs. **d** Percentages of CRC tissues with low or high expression of circRNA_0000392 according to TNM stage. **e** Analysis of circRNA_0000392 expression in CRC patients according to TNM stage. **f** Analysis of circRNA_0000392 expression in CRC patients with or without lymph node metastasis. **g** Analysis of circRNA_0000392 expression in CRC patients with or without distant metastasis. **h** Relative expression of circRNA_0000392 in cell lines was detected by qRT-PCR. Data represent the mean ± SD. * *P* < 0.05, ** *P* < 0.01

To further confirm our results, we increased the number of tissue samples to 40 pairs and detected the expression level of circRNA_0000392 by qRT-PCR. The results showed that in 29 of the 40 pairs of CRC tissues and adjacent normal tissues, the expression level of circRNA_0000392 was higher in tumor tissues than in adjacent normal tissues and was significantly increased in the overall statistics of the 40 pairs (Fig. 2a and b). Then, ROC curve analysis was performed to assess the diagnostic value of circRNA_0000392 in CRC. The results showed that circRNA_0000392 could discriminate CRC from adjacent normal tissues with an AUC of 0.713 (95% CI: 0.598–0.827, *P* < 0.01) (Fig. 2c). The associations between the circRNA_0000392 expression level and clinical parameters of the CRC patients are listed in Table 1. There was no significant difference in the expression level of circRNA_0000392 between the two groups according to age, gender, tumor size or location. However, the expression of circRNA_0000392 was significantly correlated with pathological stage (*P* = 0.0123), lymph node metastasis (*P* = 0.0055) and distant metastasis (*P* = 0.0084) (Fig. 2d–g, and Additional file 1: Fig. S4A-B). The circRNA_0000392 expression level was also measured in the normal human colon mucosal epithelial cell line NCM460 and in 7 CRC cell lines, namely, HT29, HCT116, SW480, SW837, SW48, SW620, and RKO. circRNA_0000392 expression was markedly upregulated in human CRC cell lines compared with normal colon mucosal epithelial cell lines (Fig. 2h). These results indicate that circRNA_0000392 expression was elevated in CRC and may be involved in the tumorigenesis and development of CRC.

**Table 1:** Associations between circRNA_0000392 levels and clinical parameters in colorectal cancer patients (*n* = 40)

Characteristic	No. of Patients (%)	CircRNA-0000392 expression	***P***-value (2-ΔCt Mean ± SD)
Age (y)	40 (100)		
≥60	31 (77.5)	5.458 ± 5.084	0.3699
< 60	9 (22.5)	7.247 ± 5.645	
Sex	40 (100)		
Men	27 (67.5)	5.497 ± 5.337	0.5310
Women	13 (32.5)	6.615 ± 5.010	
Tumor size (cm)	40 (100)		
≥5	18 (45)	5.839 ± 4.716	0.9810
< 5	22 (55)	5.879 ± 5.668	
Tumor location	40 (100)		
Left hemicolon	34 (85)	5.365 ± 4.880	0.1536
Right hemicolon	6 (15)	8.668 ± 6.497	
pTNM stage	40 (100)		
I-II	20 (50)	4.071 ± 3.144	0.0123^*^
III-IV	20 (50)	7.679 ± 6.294	
Lymph node metastasis	40 (100)		
pN negative	21 (52.5)	3.756 ± 3.416	0.0055^**^
pN positive	19 (47.5)	8.218 ± 5.957	
Distant metastasis	40 (100)		
pM negative	34 (85)	4.981 ± 3.995	0.0084^**^
pM positive	6 (15)	10.940 ± 8.520	

* *P* < 0.05; ** *P* < 0.01

### The characteristics of the circRNA_0000392

**Fig. 3:**
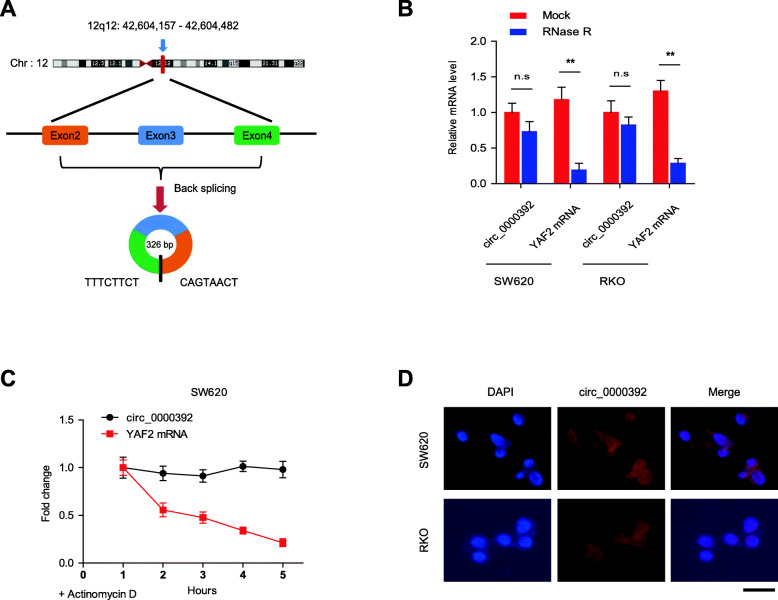
The characteristics of circRNA_0000392. **a** The genomic loci of the YAF2 gene and circRNA_0000392. The spliced mature sequence length of circRNA_0000392 is 326 bp. **b** The expression of circRNA_0000392 and YAF2 mRNA in SW620 and RKO cells treated with or without RNase R was detected by qRT-PCR. **c** qRT-PCR analysis of circRNA_0000392 and YAF2 mRNA in SW620 cells treated with actinomycin D at the indicated time points. **d** Fluorescence in situ hybridization (FISH) assays were performed to determine the localization of circRNA_0000392 in SW620 and RKO cells. Scale bar = 50 μm. Data represent the mean ± SD. * *P* < 0.05, ** *P* < 0.01

Before we delved into the specific role of circRNA_0000392 in CRC, we first investigated the characteristics of circRNA_0000392. The genomic locus of circRNA_0000392 is shown in Fig. 3a, and the spliced mature sequence length of circRNA_0000392 is 326 bp. Further, SW620 and RKO cells were treated with RNase R exonuclease and actinomycin D to verify the authenticity of circRNA_0000392. CircRNA_0000392 was resistant to RNase R (Fig. 3b) and actinomycin D (Fig. 3c and Additional file 1: Fig. S4C), whereas YAF2 mRNA was significantly reduced after RNase R and actinomycin D treatment. RNA fluorescence in situ hybridization (FISH) assays demonstrated that circRNA_0000392 was mainly localized in the cytoplasm (Fig. 3d). These data showed that the circRNA_0000392 species was indeed circular.

### Knockdown of circRNA_0000392 inhibits CRC cell proliferation and invasion

**Fig. 4:**
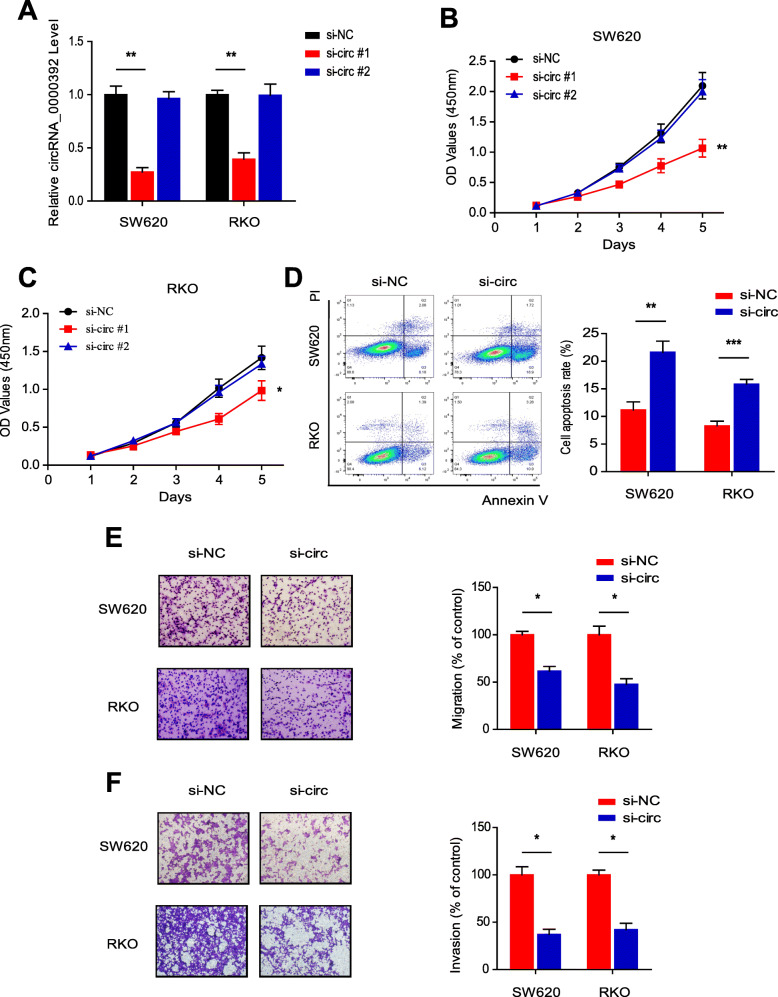
Knockdown of circRNA_0000392 inhibits CRC cell proliferation and invasion. **a** qRT-PCR analysis of circRNA_0000392 in SW620 and RKO cells after transfection with siRNA for 48 h. **b** - **c** SW620 and RKO cell proliferation after circRNA_0000392 knockdown by siRNA was detected by WST-1 assay. **d** The apoptosis rate was analyzed by flow cytometry after downregulation of circRNA_0000392 in SW620 and RKO cells. (E - F) Cell migration (**e**) and invasion (**f**) were assessed by transwell assay with or without Matrigel after circRNA_0000392 knockdown in SW620 and RKO cells. Data represent the mean ± SD. * *P* < 0.05, ** *P* < 0.01

To explore the function of circRNA_0000392 in CRC cells, we first designed two siRNAs targeting the back-splice region. Then, loss-of-function assays were performed in SW620 and RKO cells with relatively high expression of circRNA_0000392. After transfection of the two siRNAs, the expression of circRNA_0000392 was significantly reduced by siRNA #1 in both cell lines (Fig. 4a). The WST-1 assay demonstrated that downregulation of circRNA_0000392 significantly inhibited the proliferation viability of SW620 and RKO cells (Fig. 4b and c). We further investigated whether circRNA_0000392 has an effect on the apoptosis of CRC cells by flow cytometry. Double staining with Annexin V and PI showed that circRNA_0000392 knockdown significantly enhanced cell apoptosis at 48 h post transfection with siRNA #1 or si-NC (Fig. 4d). Next, cell migration and invasion abilities after siRNA transfection were performed using Transwell assays with or without Matrigel. As a result, the cell migration and invasion abilities of SW620 and RKO cells were significantly inhibited after knocking down the expression of circRNA_0000392 (Fig. 4e and f). We further measured the protein expression level of the EMT markers. As shown in Additional file 1: Fig. S9, after the expression level of circRNA_0000392 was knocked down, the expression of E-Cadherin was significantly up-regulated and the expression of Vimentin was significantly decreased. These results indicated that circRNA_0000392 contributed to CRC cell proliferation and motility in vitro.

### CircRNA_0000392 functions as a sponge for miR-193a-5p

**Fig. 5:**
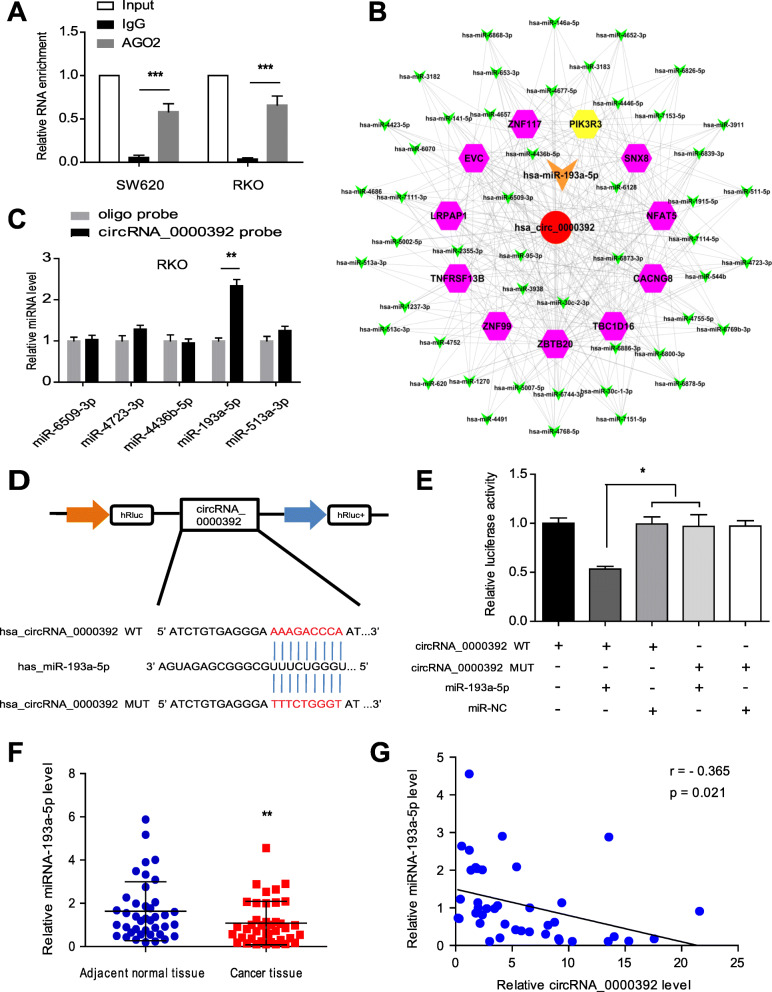
CircRNA_0000392 functions as a sponge for miR-193a-5p. **a** RIP analysis of circRNA_0000392 using anti-AGO2 antibody in SW620 and RKO cells. **b** The circRNA-miRNA-mRNA interaction based on circRNA_0000392 was demonstrated by prediction and bioinformatics analysis using Cytoscape software. **c** The top five miRNAs that may be regulated by circRNA_0000392 based on the miRNA prediction and bioinformatics analyses are shown and measured by qRT-PCR after the pull-down assay in RKO cells. **d** Schematic illustration demonstrating the luciferase reporter vectors containing wild-type (WT) or mutant (MUT) predicted miR-193a-5p binding sites of circRNA_0000392. **e** The luciferase assay was performed in 293 T cells after cotransfection with miR-193a-5p mimic and the luciferase vector containing wild-type (WT) or mutant (MUT) circRNA_0000392. **f** Relative expression of miR-193a-5p in 40 pairs of CRC and ANT tissues measured by qRT-PCR. **g** The correlation between circRNA_0000392 and miR-193a-5p in CRC tissues was analyzed by Spearman correlation coefficients. Data represent the mean ± SD. * *P* < 0.05, ** *P* < 0.01, *** *P* < 0.001

It is well known that acting as a miRNA sponge is one of the important mechanisms by which circRNAs exert their biological functions [10]. Given that we previously found by prediction software that circRNA_0000392 could function as a miRNA sponge, we mainly focused on its function as a miRNA sponge to further explore its underlying mechanism in CRC cell proliferation. First, the RIP assay was performed in SW620 and RKO cells. The results showed that circRNA_0000392 was enriched in AGO2 immunoprecipitates, confirming that circRNA_0000392 functions to adsorb miRNA (Fig. 5a). Then, circRNA-miRNA-mRNA interactions based on circRNA_0000392 were predicted by bioinformatics analysis using Cytoscape software (Fig. 5b). From the prediction results, we selected the top 5 candidate miRNAs to validate the specific interaction by RNA pull-down assay. The results showed that miR-193a-5p had a dramatic difference in the pull-down level by the circRNA_0000392 probe compared with the oligo probe in both SW620 and RKO cells (Fig. 5c and Additional file 1: Fig. S5A). To further confirm the interactions between circRNA_0000392 and miR-193a-5p, a dual-luciferase reporter assay was performed in 293 T cells. The circRNA_0000392-wt or circRNA_0000392-mut plasmid was constructed based on a luciferase reporter vector (Fig. 5d) and then cotransfected with miR-193a-5p mimic or NC in 293 T cells (Additional file 1: Fig. S5B-C). The dual-luciferase reporter assay results showed that miR-193a-5p mimics significantly reduced the luciferase activity of the circRNA_0000392-WT group but had no effect on the mutant group (Fig. 5e). The expression levels of miR-193a-5p in 40 pairs of CRC tumor tissues and adjacent normal tissues were measured by qRT-PCR. The results showed that the miR-193a-5p expression level in CRC tumor tissues was significantly reduced compared with that in adjacent normal tissues (Fig. 5f and Additional file 1: Fig. S6). Spearman correlation coefficient analysis revealed a negative correlation between miR-193a-5p and circRNA_0000392 expression in CRC tumor tissues (*r* = − 0.365, *P* = 0.021) (Fig. 5g). Overall, these results demonstrate that circRNA_0000392 acts as a sponge for miR-195-5p in CRC.

### PIK3R3 is directly targeted by miR-193a-5p and indirectly regulated by circRNA_0000392

**Fig. 6:**
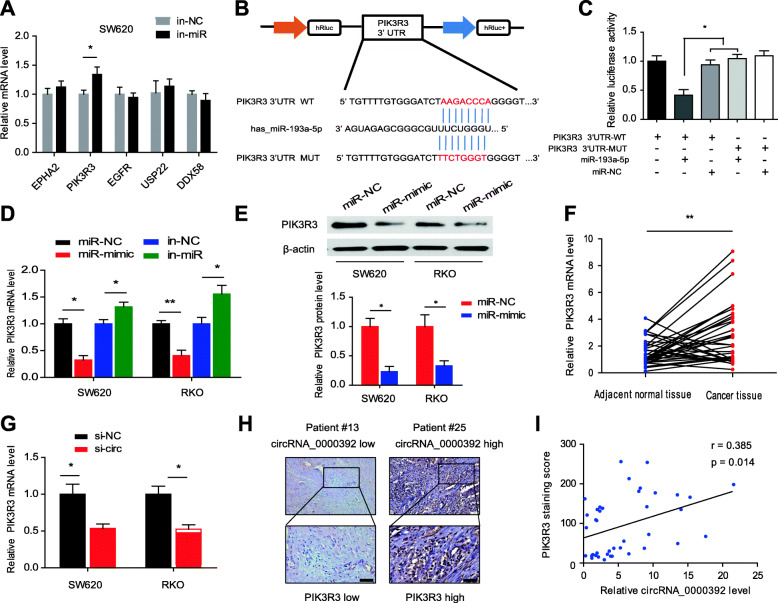
PIK3R3 is directly targeted by miR-193a-5p and indirectly regulated by circRNA_0000392. **a** The relative mRNA expression of EPHA2, PIK3R3, EGFR, USP22 and DDX58 after transfection with the miR-193a-5p inhibitor was detected in SW620 cells by qRT-PCR. **b** Schematic illustration of PIK3R3 3’UTR wild-type (WT) or 3’UTR mutant (MUT) luciferase reporter vectors and the predicted binding sites to miR-193a-5p. **c** The relative luciferase activities were detected in 293 T cells after cotransfection with the PIK3R3 3’UTR wild-type (WT) or 3’UTR mutant (MUT) luciferase reporter vectors with the miR-193a-5p mimics. **d** Relative PIK3R3 mRNA expression after transfection with the miR-193a-5p mimics or inhibitor was detected in cells by qRT-PCR. **e** The relative PIK3R3 protein level after transfection with the miR-193a-5p mimics was detected in cells by western blot. **f** Relative expression of PIK3R3 in 40 pairs of CRC and ANT tissues measured by qRT-PCR. **g** Relative PIK3R3 mRNA expression after transfection with circRNA_0000392 siRNA was detected by qRT-PCR. **h** Representative IHC staining images of low and high PIK3R3 expression in patient CRC tissue samples. Scale bar = 20 μm. **i** The correlation between circRNA_0000392 and PIK3R3 protein expression in CRC tissues was analyzed based on Spearman correlation coefficients. Data represent the mean ± SD. * *P* < 0.05, ** *P* < 0.01

According to our previous predictions, EPHA2, PIK3R3, EGFR, USP22 and DDX58 are the most likely potential target genes for miR-193a-5p. Then, we detected the mRNA expression levels of these genes after transfection of SW620 cells with the miR-193a-5p mimics or inhibitor. The results revealed that the EPHA2 and PIK3R3 expression levels were significantly downregulated by the miR-193a-5p mimic and that the PIK3R3 expression level was upregulated after transfection with the miR-193a-5p inhibitor (Fig. 6a and Additional file 1: Fig. S7A). The dual-luciferase reporter assay was performed to confirm the binding relationship between PIK3R3 and miR-193a-5p (Fig. 6b). The PIK3R3 3’UTR WT or mutant plasmid was cotransfected with the miR-193a-5p mimic in 293 T cells. The results showed that cotransfection of the PIK3R3 3’UTR WT plasmid and miR-193a-5p mimic significantly reduced the relative luciferase activity (Fig. 6c). Subsequently, we also tested whether miR-193a-5p affects the expression of PIK3R3. The qRT-PCR results revealed that the miR-193a-5p mimic could markedly reduce the expression level of PIK3R3, whereas the expression level of PIK3R3 was significantly upregulated by the miR-193a-5p inhibitor in both SW620 and RKO cell lines (Fig. 6d). PIK3R3 protein levels were significantly downregulated by intervention with the miR-193a-5p mimic (Fig. 6e). Then, we detected the expression levels of PIK3R3 in 40 pairs of CRC tumor tissues and adjacent normal tissues. The results showed that the PIK3R3 expression level in CRC tumor tissues was significantly increased compared with that in adjacent normal tissues and was negatively correlated with miR-193a-5p expression in CRC tissues (*r* = − 0.34, *P* = 0.032) (Fig. 6f and Additional file 1: Fig. S7B-D). To explore whether PIK3R3 expression levels could also be regulated by circRNA_0000392, we detected PIK3R3 expression after transfection with circRNA_0000392 siRNA. We found that knockdown of circRNA_0000392 significantly decreased the expression of PIK3R3 (Fig. 6g). Based on IHC staining of PIK3R3 in 40 CRC tissues, we found that PIK3R3 was positively correlated with circRNA_0000392 expression in CRC tissues (*r* = 0.385, *P* = 0.014) (Fig. 6h and i). Collectively, these results demonstrated that PIK3R3 was a target gene of miR-193a-5p and could be regulated by circRNA_0000392.

### CircRNA_0000392 promotes CRC cell proliferation and invasion through the circRNA_0000392/miR-193a-5p/PIK3R3 axis

**Fig. 7:**
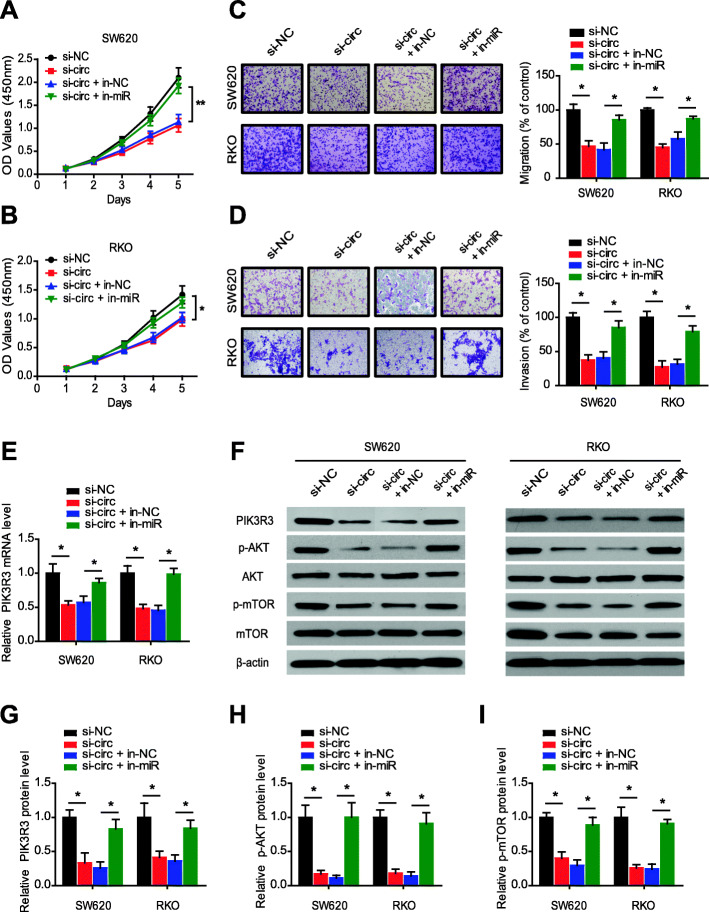
CircRNA_0000392 promotes cell proliferation and invasion through the circRNA_0000392/miR-193a-5p/PIK3R3 axis. **a** - **b** SW620 and RKO cell proliferation after transfection with circRNA_0000392 siRNA and/or miR-193a-5p inhibitor was measured by WST-1. **c** - **d** The cell migration (**c**) and invasion (**d**) capabilities were determined by Transwell assay after transfection of SW620 and RKO cells with the circRNA_0000392 siRNA and/or miR-193a-5p inhibitor. **e** The relative mRNA expression of PIK3R3 after transfection with circRNA_0000392 siRNA and/or miR-193a-5p inhibitor was detected by qRT-PCR. **f** - **i** The relative protein expression of PIK3R3 and the phosphorylation level of downstream pathway proteins were measured by western blot in cells transfected with the circRNA_0000392 siRNA and/or miR-193a-5p inhibitor. Data represent the mean ± SD. * *P* < 0.05, ** *P* < 0.01

To investigate whether circRNA_0000392 plays its role in promoting tumor progression through the/miR-193a-5p/PIK3R3 axis, rescue experiments were performed by transcription of circRNA_0000392-knockdown cells with the miR-193a-5p inhibitor. The results of WST-1 and Transwell assays indicated that the inhibition of proliferation and invasion by circRNA_0000392 knockdown in SW620 and RKO cells could be rescued by the miR-193a-5p inhibitor (Fig. 7a-d). The miR-193a-5p inhibitor also rescued the effect of circRNA_0000392 knockdown on apoptosis (Additional file 1: Fig. S8). Then, we detected PIK3R3 mRNA expression by qRT-PCR and found that the reduction in PIK3R3 expression due to circRNA_0000392 siRNA could be alleviated by the miR-193a-5p inhibitor (Fig. 7e). In addition, the western blot assay revealed that knockdown of circRNA_0000392 decreased the PIK3R3 protein levels and AKT and mTOR phosphorylation levels, and the effects could be reversed by the miR-193a-5p inhibitor (Fig. 7f-i). Collectively, these results demonstrated that circRNA_0000392 could act as a regulator of miR-193a-5p to further affect the expression of PIK3R3 and play a regulatory role in CRC.

### Downregulation of circRNA_0000392 suppresses the growth of CRC cells in vivo

**Fig. 8:**
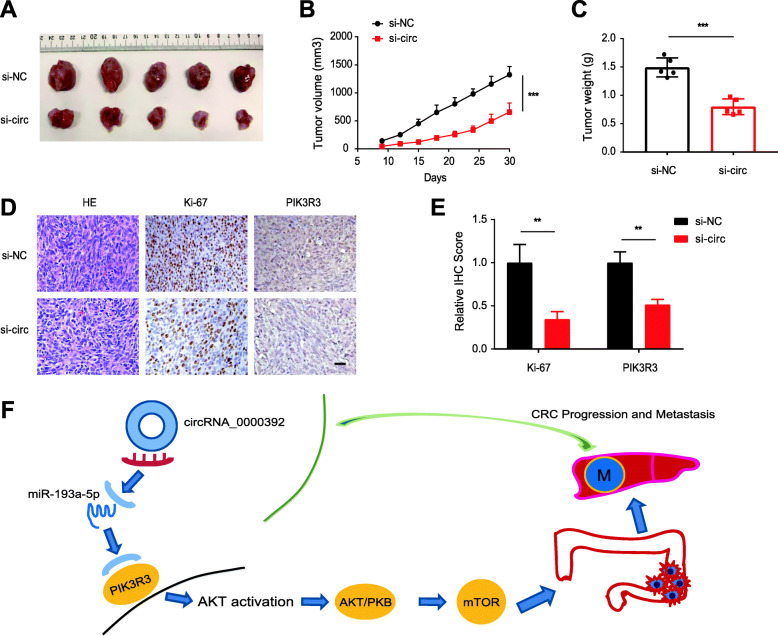
Downregulation of circRNA_0000392 suppresses the growth of CRC cells in vivo. **a** Image of subcutaneous xenograft tumors. Nude mice were injected with 5 × 10^6^ SW620 cells (*n* = 5 for each group). Tumors were extracted after 30 days. **b** Analysis of tumor volume of mice measured every 3 days. **c** Tumor weight in each group at the end of the experiment. **d** Histological analysis of tumor tissues by hematoxylin and eosin staining. IHC of Ki-67 and PIK3R3 in subcutaneous tumors. Scale bar, 100 μm. **e** The graph shows the relative signal intensity scores of KI-67 and PIK3R3. **f** Schematic illustration of circRNA_0000392 regulating the miR-193a-5p/PIK3R3 axis in CRC. Data represent the mean ± SD. * *P* < 0.05, ** *P* < 0.01, *** *P* < 0.001. Additional file 1

To explore the effects of circRNA_0000392 in vivo, circRNA_0000392-knockdown SW620 cells and negative control cells were subcutaneously injected into the backs of BALB/c nude mice. After 30 days of observation, the results showed that circRNA_0000392 knockdown reduced the volume and weight of SW620-derived tumors in vivo (Fig. 8a-c). Then, the expression levels of Ki-67 and PIK3R3 in the two groups of tumor tissues were evaluated by immunohistochemical staining. The results demonstrated that Ki-67 and PIK3R3 expression levels decreased in the tumor tissues in which the expression of circRNA_0000392 was knocked down (Fig. 8d - e). Therefore, inhibiting the expression of circRNA_0000392 significantly inhibited the growth of CRC in vivo (Fig. 8f).

## Discussion

Colorectal cancer is one of the most common malignant tumors, and its incidence has increased yearly. In terms of treatment, early CRC can be treated by endoscopic minimally invasive surgery and surgical eradication. However, given that most CRC cases have no obvious clinical symptoms in the early stage, approximately 60% of CRC patients have progressed into the middle and late stages at the time of diagnosis with lymph node and distant metastases [3]. For colorectal cancer, early detection and treatment can achieve a better prognosis, so it is important to search for effective new biomarkers and to explore CRC pathogenesis-related signaling pathways.

CircRNAs are noncoding RNAs that form a closed continuous loop by covalent attachment of the 3′ and 5′ ends [4, 18]. As early as the 1970s, Sanger et al. [19] discovered the presence of single-stranded circular RNA in plant viruses. However, due to the limitations of detection, circRNA was considered to be a phenomenon of incorrect splicing during exon transcription [20]; thus, its existence did not receive sufficient attention during that period. In recent years, with the development of high-throughput sequencing technology and bioanalysis, circRNAs have become a research hotspot in the field of biomedicine [21, 22]. CircRNAs are widely expressed in human cells and are tissue specific with varying levels of expression in different types of tissues [23]. Due to their unique characteristics, circRNAs have become promising diagnostic markers and therapeutic targets for cancer. To date, many studies have identified circRNAs as diagnostic and prognostic biomarkers in distinct human cancers [24–26] and have reported the role of circRNAs in the progression of cancers [27–30].

In our study, we performed high-throughput circRNA sequencing in cancer tissues and adjacent normal tissues of 6 colorectal cancer patients and obtained the expression profile of 66,855 circRNAs in colorectal cancer. Then, the circRNAs differentially expressed between colorectal cancer tissues and normal tissues were identified by bioinformatics analysis. These circRNAs may become potential biomarkers and therapeutic targets for the diagnosis of colorectal cancer. Based on our data, we selected some circRNAs exhibiting significant differences in expression and validated them in additional samples. We found that circRNA_0000392 was significantly upregulated in colorectal cancer tissues and cell lines. The expression level of circRNA_0000392 in colorectal cancer was markedly associated with clinical stage and malignant progression. ROC curve analysis showed the diagnostic value of circRNA_0000392 in CRC, revealing that it may be a promising prognostic biomarker. Next, a series of functional experiments demonstrated that knockdown of circRNA_0000392 significantly inhibited the proliferation and invasion of CRC cells, revealing its function as an oncogene. In particular, in the results verified by clinical samples, the expression level of circRNA_0000392 was significantly increased in the CRC lymph node and distal metastasis group. Combined with its effect on the invasion of CRC cells in vitro, it can be speculated that circRNA_0000392 plays a key role in the malignant progression of CRC.

In the research on circRNAs to date, the miRNA sponge mechanism has been one of the foundations for exploring the biological functions of circRNAs. Since Hansen [10] discovered that circRNA could function as a miRNA sponge and demonstrated that ciRS-7 acted as a miRNA sponge, numerous circRNAs with miRNA sponge function have been revealed in human cancers [15, 31, 32]. The RIP assay was used to confirm that circRNA_0000392 has can adsorb miRNA. Based on circRNA_0000392, we first predicted circRNA-miRNA-mRNA interactions through target prediction software and constructed relevant networks. We further confirmed that circRNA_0000392 could directly interact with miR-193a-5p, one of the predicted targets, by using RNA pull-down and dual luciferase reporter assays. The results of rescue experiments showed that the effect of decreased CRC cell proliferation and invasion caused by circRNA_0000392 knockdown was offset by inhibition of miR-193a-5p. It has been reported that miR-193a-5p mainly contributes as a tumor suppressor in a variety of cancers [33–35], and the interaction of circRNA_0000392 with miR-193a-5p attenuates the tumor suppressor efficiency of miR-193a-5p. Our results demonstrated that circRNA_0000392 acts as an oncogene by sponging miR-193a-5p in CRC.

As noncoding RNAs, miRNAs exert biological effects by modulating their target genes. The circRNAs with miRNA sponge function can adsorb miRNAs and indirectly release the inhibitory effects of miRNAs on their targeted genes. After we determined that miR-193a-5p could be adsorbed by circRNA_0000392, our next focus was to search for its effector target genes. Similarly, from the prediction analysis, we selected several of the most likely potential target genes and experimentally determined that miR-193a-5p could specifically bind to the 3′ UTR of PIK3R3 and regulate its expression level.

PI3K signaling is widely activated in human cancers, and its role in tumor development and metastasis has been well investigated. PIK3R3 is one of the mammalian genes from Class IA PI3Ks and encodes the p85a, p85b and p55g regulatory subunits [36]. The PIK3R3 regulatory subunit is important for cell proliferation and tumorigenesis [37]. Additionally, PIK3R3 is overexpressed in some cancers and has been reported to act as an oncogene. Our data showed that PIK3R3 mRNA and protein expression levels in CRC tissues were elevated compared with those in adjacent normal tissues and had a significant positive correlation with the expression level of circRNA_0000392. Studies have shown that PIK3R3 expression levels in CRC and ovarian cancer tissues exhibit the same trend, which is consistent with our results [38, 39]. Our in vitro results showed that knocking down the expression of circRNA_0000392 inhibited CRC cell proliferation and invasion, whereas the expression level of PIK3R3 and phosphorylation levels of AKT1 and mTOR were also inhibited. Subsequently, rescue experiments also showed that the miR-193a-5p inhibitor restored the inhibitory effect of knocking down circRNA_0000392 on cell proliferation and restored the inhibition of this pathway by downregulation of circRNA_0000392. Abnormal activation of AKT-mTOR signaling pathway plays an important role in the malignant progression of CRC [40–42]. Consistently, our results showed that downregulation of circRNA_0000392 significantly inhibit the phosphorylation level of AKT (Ser473). CircRNA_0000392 serves as a regulator of AKT/mTOR signaling in CRC cells. Thus, the circRNA_0000392/miR-193a-5p/PIK3R3/AKT axis plays an important role in CRC.

## Conclusion

In conclusion, we revealed that circRNA_0000392 was upregulated in human CRC tissues and is a promising biomarker for CRC. Furthermore, we first demonstrated the effect of the circRNA_0000392/miR-193a-5p/PIK3R3 axis on the activation of the AKT-mTOR pathway, representing a novel mechanism for CRC progression. This study suggests that circRNA_0000392 is a potential therapeutic target for the treatment of colorectal cancer and a predictive marker for CRC patients.

## Supplementary Information

**Additional file 1: Figure S1.** GO analyses of the host genes of differentially expressed circRNAs. (A-B) GO annotations of the host genes of significantly upregulated expressed circRNAs. **(C-D)** GO annotations of the host genes of significantly downregulated expressed circRNAs. **Figure S2.** KEGG analyses of the host genes of differentially expressed circRNAs. (A-B) KEGG analyses of the host genes of significantly upregulated expressed circRNAs. **(C-D)** KEGG analyses of the host genes of significantly downregulated expressed circRNAs. **Figure S3.** CircRNA-miRNA network maps were constructed using Cytoscape software based on significant differential expression of circRNAs to demonstrate their interaction. **Figure S4.** Analysis of circRNA_0000392 expression in CRC patients with or without lymph node metastasis (A) and CRC patients with or without distant metastasis (B). (C) qRT-PCR analysis of circRNA_0000392 and YAF2 mRNA in RKO cells treated with actinomycin D at the indicated time points. **Figure S5.** (A) The top five miRNAs may regulated by circRNA_0000392 base on the miRNA prediction and bioinformatics analyses were showed and measured by qRT-PCR after the pull – down assay in SW620 cells. (B) Relative expression of miR-193a-5p after transfected with the mimics or inhibitor was measured by qRT-PCR. **(C)** Relative expression of circRNA_0000392 after transfected with the mimics or inhibitor was measured by qRT-PCR. **Figure S6.** (A-B) Relative expression of miR-193a-5p in 40 pairs of CRC and ANT tissues measured by qRT-PCR. **Figure S7.** (A) The relative mRNA expression of EPHA2, PIK3R3, EGFR, USP22 and DDX58 after transfected with the miR-193a-5p mimics was detected in SW620 cells by qRT-PCR. (B-C) Relative mRNA expression of PIK3R3 in 40 pairs of CRC and ANT tissues measured by qRT-PCR. (D) The correlation between miR-193a-5p and PIK3R3 in CRC tissues was analyzed by Spearman correlation coefficients. **Figure S8.** Apoptosis rate was analyzed by flow cytometry after transfection the indicated plasmids in SW620 and RKO cells. **Figure S9.** The relative protein expression of E-Cadherin and Vimentin were measured by western blot in cells transfected with the circRNA_0000392 siRNA or negative control. **Table S1.** Primers and RNA sequences used in this study. **Table S2.** The top 10 upregulated circRNAs and top 10 downregulated circRNAs from the circRNA-seq.

**Additional file 2.** Supplementary Materials and Methods.

**Additional file 3.** CircRNA Expression Profiling in this study (please see the attached excel spreadsheet).

**Additional file 4.** Differentially Expressed circRNAs between the two groups (please see the attached excel spreadsheet).

## Data Availability

The datasets used during the current study are available from the corresponding authors upon reasonable request.

## Abbreviations

CRC: Colorectal cancer
circRNA: Circular RNAs
qRT-PCR: Real-time quantitative polymerase chain reaction
IHC: Immunohistochemistry
RIP: RNA immunoprecipitation
ncRNA: Noncoding RNAs
PIK3R3: Phosphoinositide-3-Kinase Regulatory Subunit 3
ROC: Receiver operating characteristic
AUC: Area under the curve
EPHA2: EPH Receptor A2
EGFR: Epidermal Growth Factor Receptor
USP22: Ubiquitin Specific Peptidase 22
DDX58: DExD/H-Box Helicase 58
