# Supplementary material for: CircRNA_0000392 promotes colorectal cancer progression through the miR-193a-5p/PIK3R3/AKT axis
Source: J Exp Clin Cancer Res. 2020 Dec 14;39:283. doi: 10.1186/s13046-020-01799-1 (PMC7735421; doi:10.1186/s13046-020-01799-1)
Supplement: Supplementary file 1 — Additional file 1: Figure S1. GO analyses of the host genes of differentially expressed circRNAs. (A-B) GO annotations of the host genes of significantly upregulated expressed circRNAs. (C-D) GO annotations of the host genes of significantly downregulated expressed circRNAs. Figure S2. KEGG analyses of the host genes of differentially expressed circRNAs. (A-B) KEGG analyses of the host genes of significantly upregulated expressed circRNAs. (C-D) KEGG analyses of the host genes of significantly downregulated expressed circRNAs. Figure S3. CircRNA-miRNA network maps were constructed using Cytoscape software based on significant differential expression of circRNAs to demonstrate their interaction. Figure S4. Analysis of circRNA_0000392 expression in CRC patients with or without lymph node metastasis (A) and CRC patients with or without distant metastasis (B). (C) qRT-PCR analysis of circRNA_0000392 and YAF2 mRNA in RKO cells treated with actinomycin D at the indicated time points. Figure S5. (A) The top five miRNAs may regulated by circRNA_0000392 base on the miRNA prediction and bioinformatics analyses were showed and measured by qRT-PCR after the pull – down assay in SW620 cells. (B) Relative expression of miR-193a-5p after transfected with the mimics or inhibitor was measured by qRT-PCR. (C) Relative expression of circRNA_0000392 after transfected with the mimics or inhibitor was measured by qRT-PCR. Figure S6. (A-B) Relative expression of miR-193a-5p in 40 pairs of CRC and ANT tissues measured by qRT-PCR. Figure S7. (A) The relative mRNA expression of EPHA2, PIK3R3, EGFR, USP22 and DDX58 after transfected with the miR-193a-5p mimics was detected in SW620 cells by qRT-PCR. (B-C) Relative mRNA expression of PIK3R3 in 40 pairs of CRC and ANT tissues measured by qRT-PCR. (D) The correlation between miR-193a-5p and PIK3R3 in CRC tissues was analyzed by Spearman correlation coefficients. Figure S8. Apoptosis rate was analyzed by flow cytometry after transfection the indi [file 13046_2020_1799_MOESM1_ESM.pdf]

Supplementary Figures

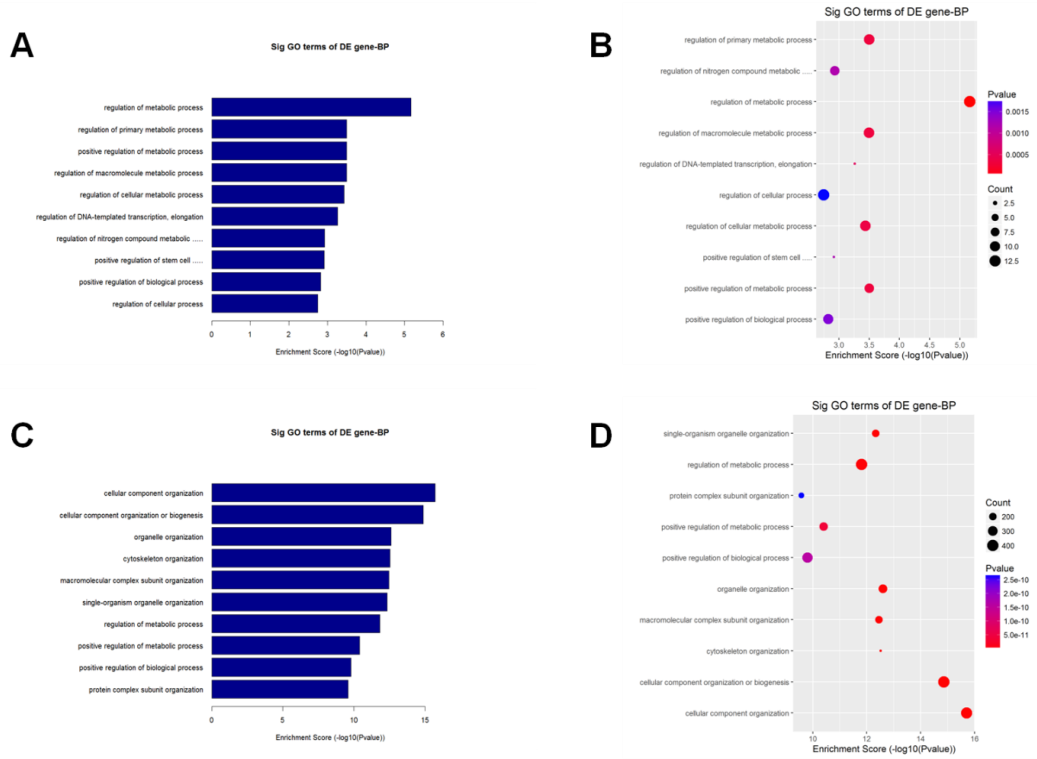

Supplementary Fig. 1. GO analyses of the host genes of differentially expressed circRNAs. (A-B) GO annotations of the host genes of significantly upregulated expressed circRNAs. **(C-D)** GO annotations of the host genes of significantly downregulated expressed circRNAs.

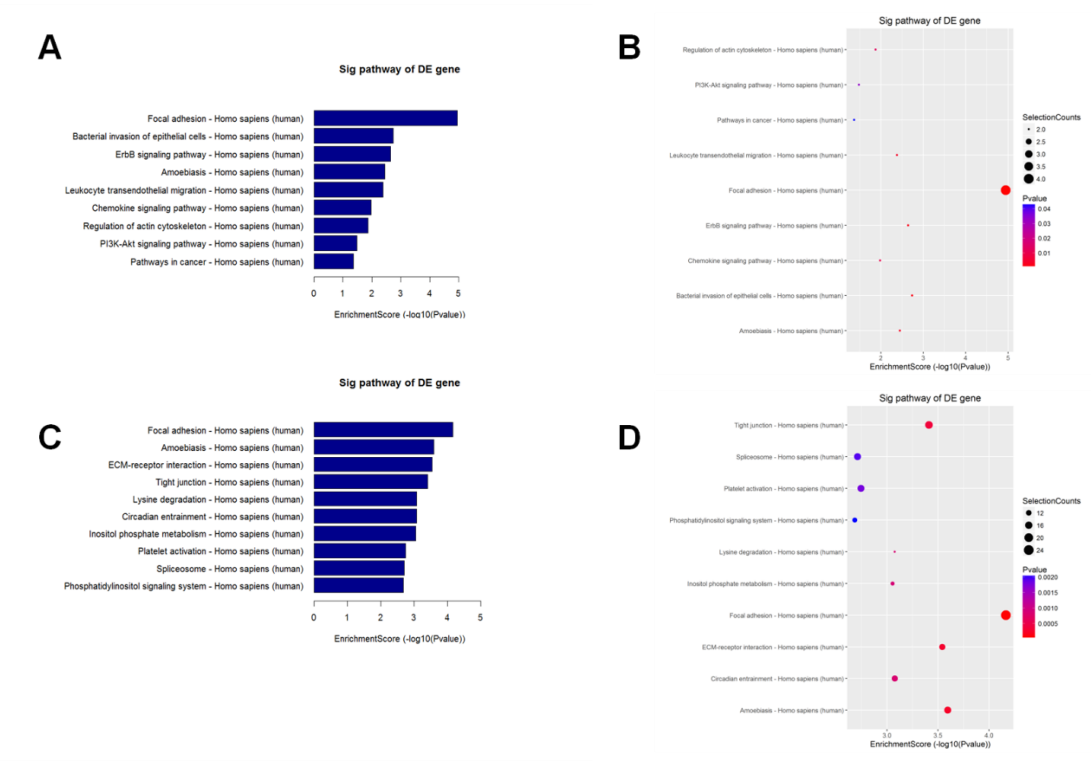

Supplementary Fig. 2. KEGG analyses of the host genes of differentially expressed circRNAs. (A-B) KEGG analyses of the host genes of significantly upregulated expressed circRNAs. **(C-D)** KEGG analyses of the host genes of significantly downregulated expressed circRNAs.

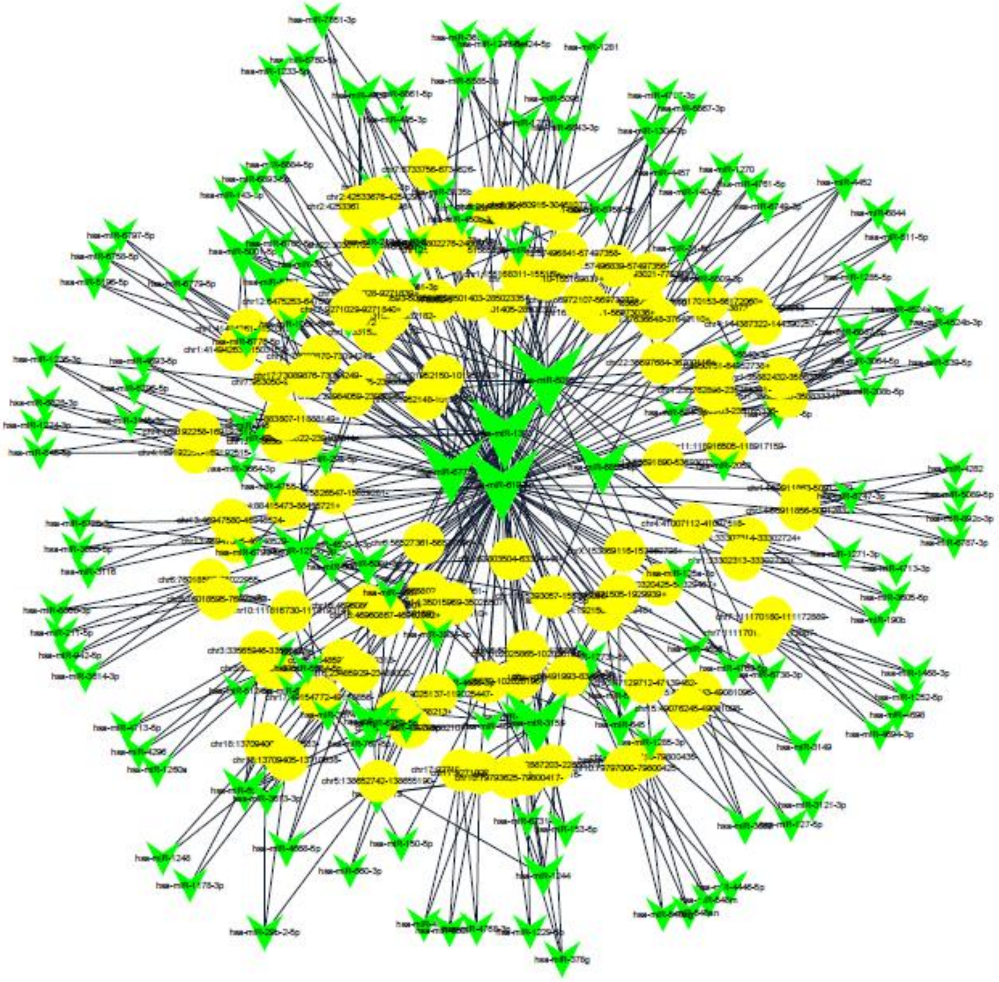

Supplementary Fig. 3. CircRNA-miRNA network maps were constructed using Cytoscape software based on significant differential expression of circRNAs to demonstrate their interaction.

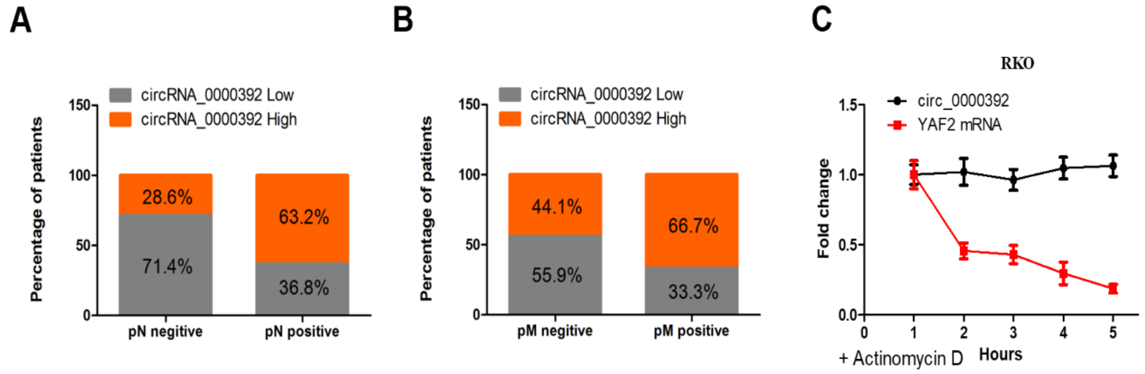

Supplementary Fig. 4. Analysis of circRNA_0000392 expression in CRC patients with or without lymph node metastasis (A) and CRC patients with or without distant metastasis (B). (C) qRT-PCR analysis of circRNA_0000392 and YAF2 mRNA in RKO cells treated

with actinomycin D at the indicated time points.

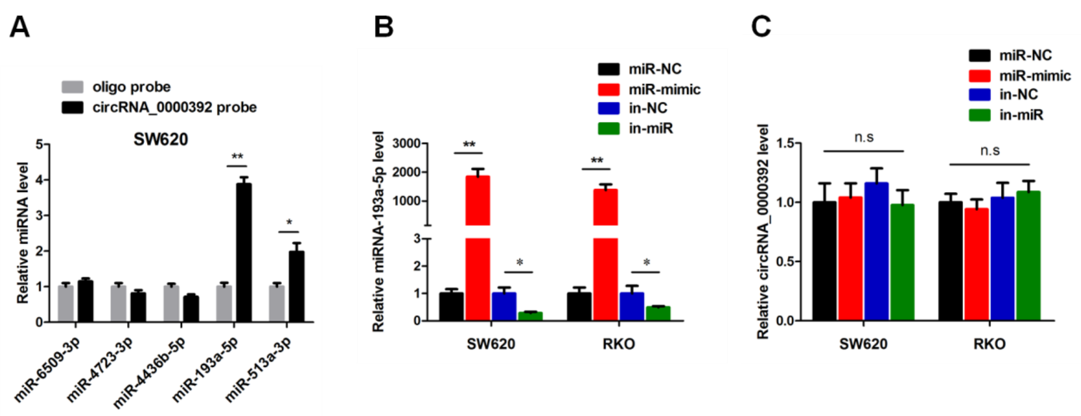

Supplementary Fig. 5. (A) The top five miRNAs may regulated by circRNA_0000392 base on the miRNA prediction and bioinformatics analyses were showed and measured by qRT-PCR after the pull – down assay in SW620 cells. **(B)** Relative expression of miR-193a-5p after transfected with the mimics or inhibitor was measured by qRT-PCR. **(C)** Relative expression of circRNA_0000392 after transfected with the mimics or inhibitor was measured by qRT-PCR.

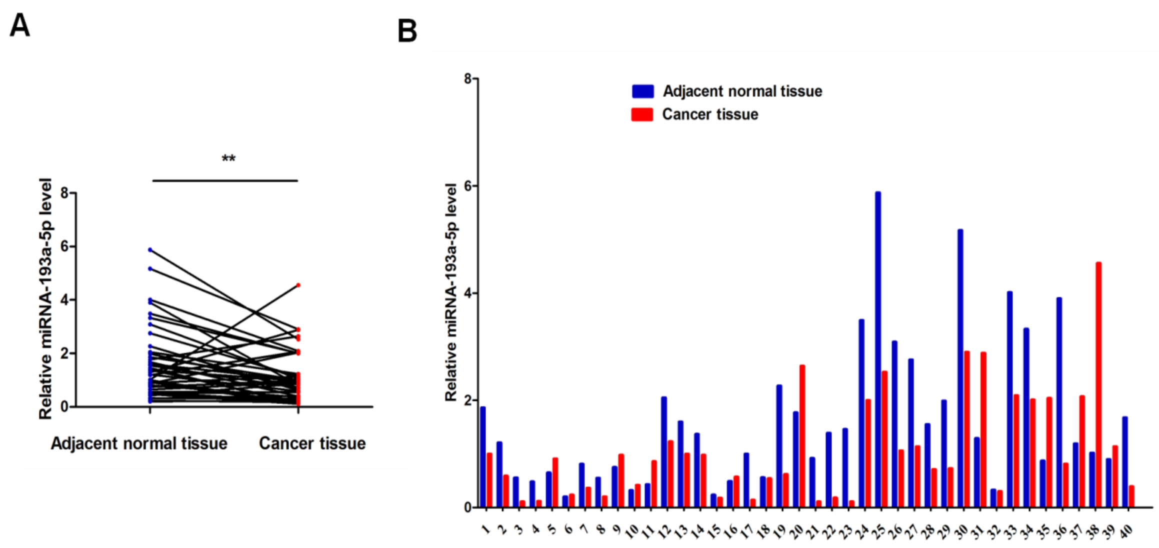

Supplementary Fig. 6. (A-B) Relative expression of miR-193a-5p in 40 pairs of CRC and ANT tissues measured by qRT-PCR.

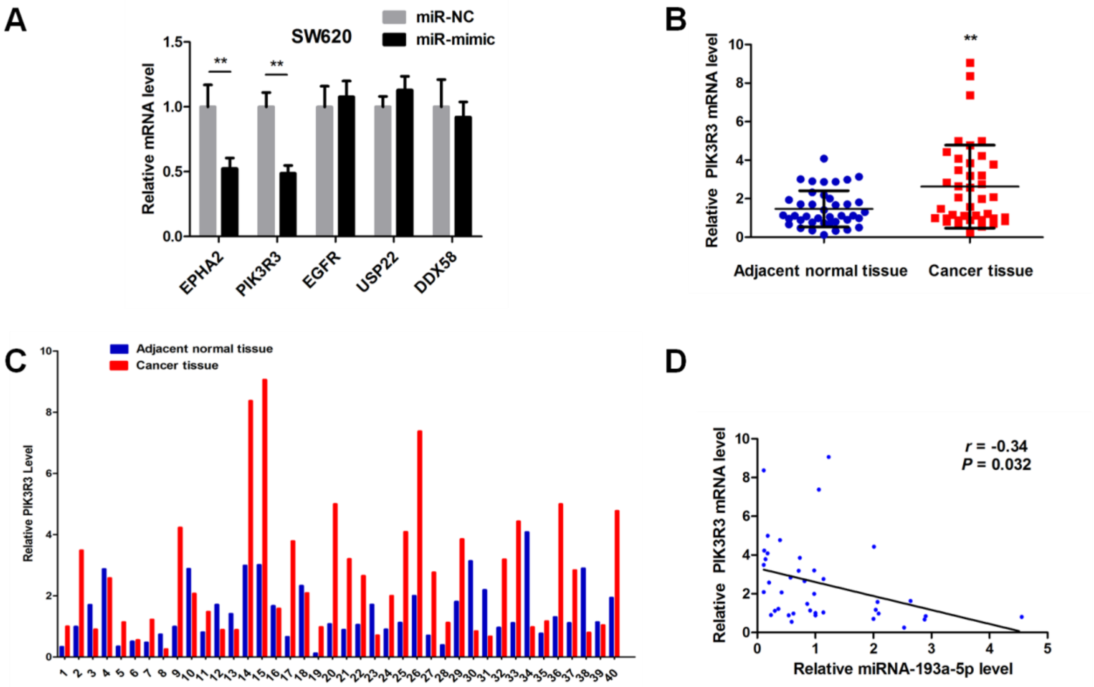

Supplementary Fig. 7. (A) The relative mRNA expression of EPHA2, PIK3R3, EGFR, USP22 and DDX58 after transfected with the miR-193a-5p mimics was detected in SW620 cells by qRT-PCR. **(B-C)** Relative mRNA expression of PIK3R3 in 40 pairs of CRC and ANT tissues measured by qRT-PCR. **(D)** The correlation between miR-193a-5p and PIK3R3 in CRC tissues was analyzed by Spearman correlation coefficients.

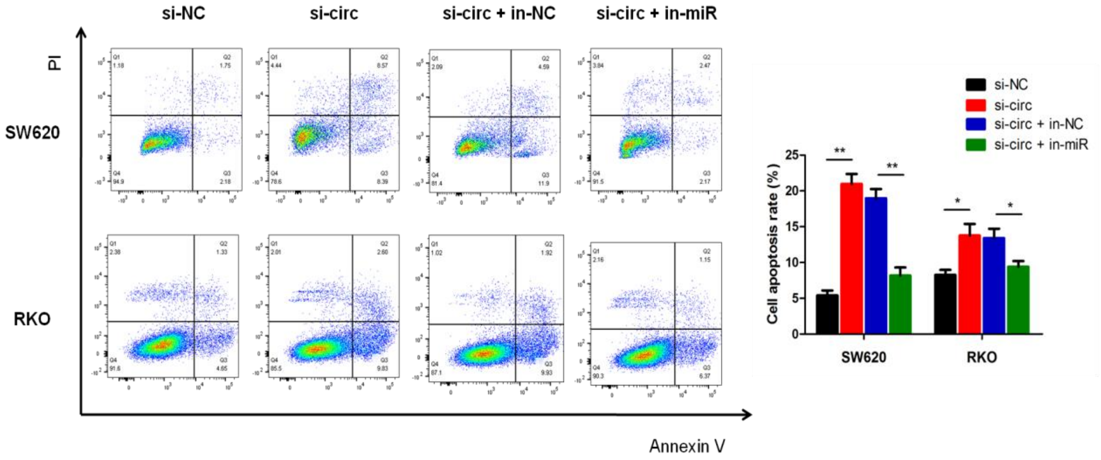

Supplementary Fig. 8. Apoptosis rate was analyzed by flow cytometry after transfection the indicated plasmids in SW620 and RKO cells.

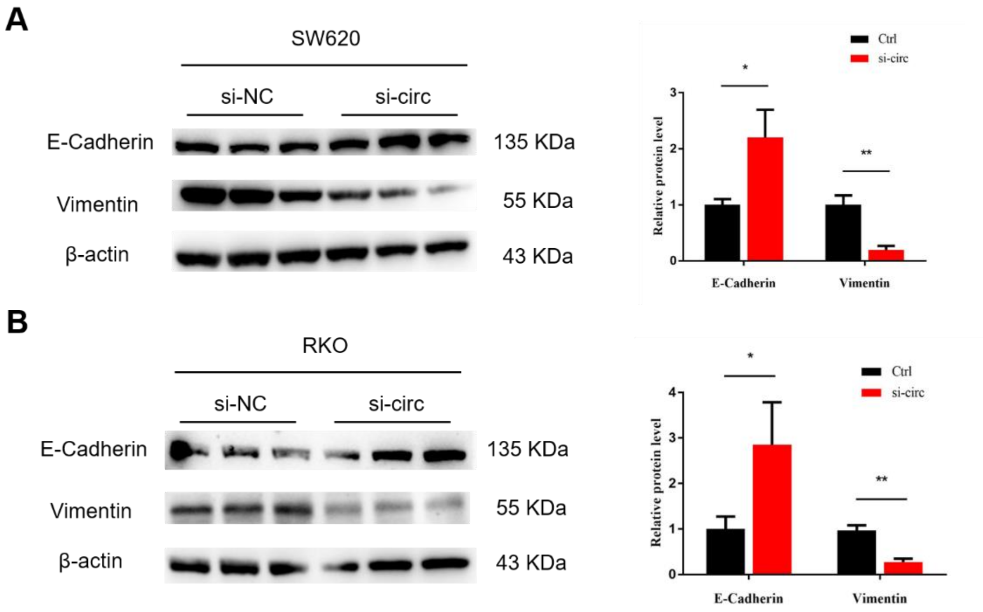

Supplementary Fig. 9. The relative protein expression of E-Cadherin and Vimentin were measured by western blot in cells transfected with the circRNA_0000392 siRNA or negative control.

Supplementary Tables

Supplementary Table 1. Primers and RNA sequences used in this study

Gene name	Forward primer	Reverse primer
hsa_circ_0000392	GCTGAACAGAAGGGCAAGAG	TTGGAAATTTCAACGGTTCC
hsa_circ_0001535	CAATGAAGCTATGCAGCAAGA	CACACCACACTTTGCTGTTGTA
hsa_circ_0003007	GCTTTTCTGCCCTCAGACAC	GGTTGGAACCCAACTACCTG
hsa_circ_0002483	GATATGGGCGTCTCCAGTGT	GCAGCTGCCATTATTTTGCT
β-actin	GTGGCCGAGGACTTTGATTG	CCTGTAACAACGCATCTCATATT
YAF2	ATGAGGGTTACTGGGACTGTAG	CCTTCCGCACATCGCACAT
EPHA2	AGAGGCTGAGCGTATCTTCAT	GGTCCGACTCGGCATAGTAGA
PIK3R3	TACAATACGGTGTGGAGTATGGA	TCATTGGCTTAGGTGGCTTTG
EGFR	TTGCCGCAAAGTGTGTAACG	GTCACCCCTAAATGCCACCG
USP22	GGAAAATGCAAGGCGTTGGAG	GTGCAGTTCGAGGTGATCTTT
DDX58	TGTGCTCCTACAGGTTGTGGA	CACTGGGATCTGATTCGCAAAA
miR-193a-5p	TATATGGGTCTTTGCGGGCG	GTGCAGGGTCCGAGGT
U6	CGCAAGGATGACACGCAA	GTGCAGGGTCCGAGGT
**miRNA mimics and inhibitors**		
hsa-miR-193a-5p mimics sense	UGGGUCUUUGCGGGCGAGAUGA	
hsa-miR-193a-5p mimics anti-sense	AUCUCGCCCGCAAAGACCCAUU	
hsa-miR-193a-5p inhibitor	UGGGUCUUUUUUGCGGGCGAGAUG	
**siRNA**		

Si-hsa_circ_0000392-1 sense	CUCAAGUUACUGAGAAGAATT
Si-hsa_circ_0000392-1 anti-sense	UUCUUCUCAGUAACUUGAGTT
Si-hsa_circ_0000392-2 sense	GUUACUGAGAAGAAAAAGCTT
Si-hsa_circ_0000392-2 anti-sense	GCUUUUUCUUCUCAGUAACTT

Supplementary Table 2. The top 10 upregulated circRNAs and top 10 downregulated circRNAs from the circRNA-seq

CircRNA ID	CircBase ID	Gene symbol	Fold changes	*P*-value
chr12:42604157-42604482-	circRNA_0000392	YAF2	24.7743759	0.0045230371643
chr13:33091994-33101669-	circRNA_0000471	N4BP2L2	13.5247731	0.0069716912671
chr14:32559708-32563592+	circRNA_0031583	ARHGAP5	12.9699781	0.0068609932778
chr19:49994751-49995003+	NA	RPL13A	9.2143207	0.0385458492475
chr11:65267947-65268121+	NA	MALAT1	7.6726098	0.00347182725
chr5:137320946-137324004-	circRNA_0001535	FAM13B	7.5234613	0.0243299719178
chr20:57014001-57016139+	circRNA_0001173	VAPB	5.9031048	0.0214436499341
chr15:101104897-101105470-	circRNA_0003007	LINS	5.4840268	0.0291610480461
chr3:195415404-195416309+	circRNA_0008267	LINC00969	5.2272882	0.0326334735724
chr8:141874411-141900868-	NA	DNAJC3	5.1256745	0.0461082775399
chr6:131247745-131277639-	circRNA_0077837	EPB41L2	-16.5373925	0.0160833348308
chr21:9826186-9826580+	NA	MIR3687-1	-12.4397176	0.0331224378607
chr5:76758920-76760634-	circRNA_0001498	WDR41	-9.1724693	0.0310305925516

chr14:50320581-50329515-	NA	Metazoa_SRP	-8.5730967	0.0149285105264
chr8:131370263-131374017-	circRNA_0085616	ASAP1	-6.556057	0.0184800508094
chr8:68117578-68118947-	NA	ARFGEF1	-5.4591184	0.0438781345434
chr11:65267861-65268260+	NA	MALAT1	-5.4591184	0.0428605568472
chr7:24663285-24720139+	circRNA_0001687	MPP6	-4.8635216	0.0485052074575
chr11:65267937-65268108-	NA	XLOC_009474	-4.4665897	0.0487781568928
chr11:65267941-65268112-	NA	XLOC_009474	-4.4665897	0.0487781568928
