# Supplementary material for: CircRNA_0000392 promotes colorectal cancer progression through the miR-193a-5p/PIK3R3/AKT axis
Source: J Exp Clin Cancer Res. 2020 Dec 14;39:283. doi: 10.1186/s13046-020-01799-1 (PMC7735421; doi:10.1186/s13046-020-01799-1)
Supplement: Supplementary file 2 — Additional file 2. Supplementary Materials and Methods. [file 13046_2020_1799_MOESM2_ESM.docx]

**Supplementary Materials and Methods**

**Cell transfection**

The SW620 and RKO cells were transfected with indicated siRNA, microRNA mimics or inhibitors (GenePharma, Shanghai, CHN) using the Lipofectamine 2000 (Invitrogen, Carlsbad, CA) according to the manufacturer’s recommendations (20pmol siRNA or 2umol mimics/inhibitors with 10ul Lipo 2000). Cells were harvested 48 hours after transfection.

**Western blot analysis**

The total proteins were extracted with RIPA buffer and quantified using BCA Protein assay kit (Thermo Scientific, USA). Then electrophoresis and electrotransfected onto a PVDF membrane (Bio-Rad, CA, USA). The membranes were blocked with 5% skimmed milk powder and incubated with primary antibodies at 4 °C overnight. Then wash and incubated with secondary antibodies (1:5000) (Santa Cruz Biotechnology USA) at room temperature for 1 h. Finally, the relevant protein was visualized by enhanced chemiluminescence system (PerkinElmer) according to the manufacturer’s instructions.

**Cell proliferation assay**

For the cell proliferation assay, SW620 and RKO cells transfected with the indicated plasmids were seeded into 96-well plates, and cell viability was assessed by premix WST-1 cell proliferation assay (TaKaRa, Japan). The absorbance of each well was read at a wavelength of 450 nm on a spectrophotometer.

**Cell apoptosis assay**

Cell apoptosis assay was implemented in SW620 and RKO cells after transfection. The cells were double stained with fluorescein isothiocyanate (FITC)-conjugated Annexin V and propodium iodide (PI). Then, the percentage of early apoptotic cells was analyzed on a flow cytometer ( BD FACSCanto, USA).
